# Molecular Dynamics Investigation of the Deformation Mechanism of Gold with Variations in Mold Profiles during Nanoimprinting

**DOI:** 10.3390/ma14102548

**Published:** 2021-05-14

**Authors:** Abhaysinh Gaikwad, Salil Desai

**Affiliations:** Center for Excellence in Product Design and Advanced Manufacturing, North Carolina A & T State University, Greensboro, NC 27411, USA; ahgaikwa@aggies.ncat.edu

**Keywords:** deformation mechanism, gold, lattice dislocations, nanoimprint lithography, molecular dynamics

## Abstract

Understanding the deformation behavior during nanoimprint lithography is crucial for high resolution patterning. Molecular dynamics modeling was implemented to investigate the effect of different mold profiles (cylindrical, rectangular, and spherical) on the von Mises stress, lattice dislocations, and material deformation. Relatively higher von Mises stress (1.08 × 10^7^ Pa) was observed for the spherical mold profile compared to the rectangular and cylindrical profiles due to the larger surface area of contact during the mold penetration stage of NIL. Substantial increases in the von Mises stress were observed for all the mold geometries during the mold penetration stage. The von Mises stresses had a reduction in the relaxation and mold retrieval stages based on the rearrangement of the gold atoms. The lattice dislocation during the deformation process revealed the formation of the BCC structure which further reverted to the FCC structure after the mold retrieval. The polyhedral template matching (PTM) method was used to explain the retention of the FCC structure and subsequent ductile behavior of the substrate. The cylindrical mold had the lowest percentage spring back in both of the orthogonal directions and thus replicated the mold profile with high-fidelity as compared to the spherical and rectangular molds. The findings of this research can aid the design of molds for several applications.

## 1. Introduction

Nanoimprint lithography (NIL) is a promising technology for fabricating nanoscale features with high precision and at lower costs. [1]. Nanoimprint lithography is one of the best top down nanomanufacturing techniques which works on the principle of deformation of substrate material to form nanoscale patterns or structures [2]. NIL creates nanoscale structures in temperature, time, and pressure-controlled environment by stepwise physical deformation of a soft material [3]. In this technique, polymer resists are uniformly spread on a silicon wafer substrate using the spin coating method. Then, a mold with precise nanoscale patterns is pressed against the substrate. The mold is pulled back and the nanopatterns on the mold are imprinted on to the resist [4]. This eliminates the limitations of traditional optical lithography which is constrained by the wavelength of the exposed UV light for fabricating nanoscale features [3,5]. In recent times, the principle of nanoimprint lithography has been used to directly form nanoscale patterns of metal films and metal nanoparticles. Metallic nanopatterns have been applied in areas such as photonics with nano-gratings, electromagnetic relays, and nanoscale biomedical devices [6,7]. Traditionally, metal nanopatterning encompasses multiple phases; the initial phase is lithography to produce nanostructures followed by the next phase of secondary operations such as electroplating to deposit a metal layer on the nanostructures and the final phase of finishing operations such as lift-off or etching. This traditional process of manufacturing metal nanopatterns is time consuming and costly [8]. Moreover, the post-processing operations add to the overhead costs and lead time, negatively impacting practical applications of nanoscale features.

In order to understand the nanoimprinting process, Sun and his team utilized the Finite Element Method (FEM) for studying deformation of a polymer in the nanoimprint lithography process [9]. Another group of researchers explored mold breakages in nanoimprint lithography of a polymer using the finite element method [10]. In the past, researchers employed molecular dynamics to analyze the temperature variations, force interactions, mold taper angle, and deformation mechanism during the different stages of nanoimprint lithography of polymers as well as metals [11,12,13]. Polymers such as Polymethyl Methacrylate (PMMA), polystyrene, and metals namely copper, aluminum, gold were used as a substrate material for these studies [14,15,16,17,18]. The thermal nanoimprint lithography process has been studied in both metallic and polymer systems using molecular dynamics simulations [19]. Further, predictive models have been developed to understand the correlation between input factors and imprint quality [20,21].

However, the limitation of FEM studies is that they are not able to explain the atomistic mechanisms at the nanoscale as they illustrate the collective performance of atoms at the continuum scale. Molecular dynamics modeling is a very accurate and advanced method to understand the atomistic phenomenon of the nanoimprinting process. It comprises of atomistic interaction of molecules in the system with each other and the interaction of system molecules with their environment [22,23]. Molecular dynamics is an efficient alternative to the finite element method for studying direct metal nanoimprinting. Atomistic simulations have been implemented to study nanoscale phenomena that capture the thermodynamics, biological phenomena, and material deformation behavior [24,25,26,27,28].

Gold is an attractive material for the nanoimprint process due to its exceptional chemical durability, thermodynamic properties, corrosion resistance, and applicability for forming processes [29,30,31]. However, performing experiments with gold substrate is an expensive venture so, utilizing molecular dynamics simulations can potentially save significant amounts of resources. In our previous effort using molecular dynamics, we discovered that the depth of mold insertion has a significant impact on the spring back effect and density variation in the substrate [32]. In another paper, using molecular dynamics, we showed that the cooling time during nanoimprint lithography of gold has a substantial impact on the resolution of final nanoimprints [29]. Moreover, we displayed that mold velocity and imprint temperature contribute towards the accuracy and quality of the final nanoimprints during nanoimprint lithography [33].

Exploring the atomistic deformation process of gold substrate by silicon mold during direct nanoimprinting is crucial for higher fidelity nanopattern imprints. Although there have been studies revealing the deformation mechanism in direct metal nanoimprinting, the molecular deformation mechanism with respect to different types of mold geometries has been underexplored. To our knowledge, there have been no molecular dynamics studies directed to investigate the outcome of different mold geometries such as cylindrical, spherical, and rectangular on direct nanoimprinting of gold. The near-spherical mold pattern was chosen because typical hemispherical molds cannot reproduce a perfect negative replica within the substrate due to spring-back effects.

In this work, we studied the effect of varying mold nanopatterns on the von Mises stress and dislocation dynamics of physical deformation of gold substrate during the nanoimprinting process. The von Mises stress profiles reveal regions of stress concentration, which can be minimized to reduce the defects in nanopattern imprints. Furthermore, dislocation of the lattice structure of gold substrate can assist in maintaining its ductile nature and thus formability. Unlike polymeric resists, direct imprinting of gold nanostructures provides immediate applications in photonics, biosensors, optical gratings, and heat exchanger membranes. The high malleability of gold and its inert nature has proven to be an asset for practical devices [34]. In our current work, the process parameters were held constant such as the imprint temperature at 473 K because this is the recrystallization temperature of gold. At 473 K, gold undergoes recrystallization and softens thereby exerting less reaction on the mold during the insertion phase [35]. The mold velocity of 50 m/s was kept constant for the molding and demolding phase with minimal internal stresses after high strain deformation of NIL. This research provides valuable design guidelines for a variety of nanoimprinting molds for direct nanoimprinting of gold substrate.

## 2. Materials and Methods

The Large-scale Atomic/Molecular Massively Parallel Simulator (LAMMPS) open source code [36] was implemented to execute the molecular dynamics simulations. LAMMPS permits parallel computations for a variety of force fields across biological, metallic, ceramic, and composite materials. The simulations were visualized using the Open Visualization Tool (OVITO) platform where results were analyzed for different time steps. [37]. The Extreme Science and Engineering Discovery Environment (XSEDE) resource provided by the National Science Foundation was utilized to conduct the simulations given its high-speed parallel performance [38].

**Figure 1:**
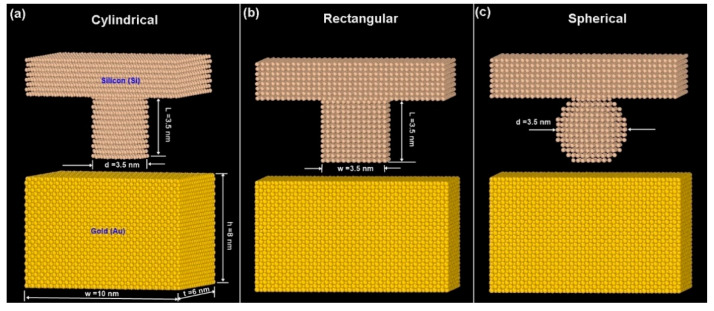
Different mold patterns with dimensions. (**a**) Cylindrical (**b**) Rectangular (**c**) Spherical.

The substrate was modeled with 30,000 gold atoms with face centered cubic (FCC) crystal structure and having dimensions of (width ‘w’) 10 nm × (height ‘h’) 8 nm × (thickness ‘t’) 6 nm as shown in Figure 1. The mold was designed using silicon with cylindrical, rectangular, and spherical profiles. The lattice structure used for the silicon mold was face centered cubic (FCC) with an orientation of (111) that exists with imprint lithography molds prepared by crystallographic wet etching [39,40]. Silicon molds are typically fabricated from wafers with crystallographic orientations of (111) due to the high anisotropic etching properties along the (110) with electron beam machining [41]. Wherein, the cubic unit cell has an edge length = 1.0. The edge vectors of the unit cell are a1 = 1 0 0, a2 = 0 1 0, and a3 = 0 0 1. Figure 1 depicts the three types of mold patterns with cylindrical, rectangular, and spherical mold tooth profile with the same aspect ratio (AR = 1) considered for the simulations. Aspect ratio was calculated as a ratio of the mold length (L) to its width (w) or diameter (d) dimension for all the molds; in the case of rectangular mold (L/w), cylindrical mold (L/d), and spherical (d/d). The cylindrical, rectangular, and spherical profile was composed of 5312, 5454, and 5151 silicon atoms, respectively. Several photonic and optical applications involve the use of nanoscale hemispherical dimples on substrate surfaces for CMOS sensor arrays and GaN-based LEDs [42,43]. A near-spherical shaped mold was chosen to compensate for the spring-back effect that occurs with conventional hemispherical molds. Typical hemispherical molds cannot reproduce deeper cavities in metals resulting in non-optimal and shallow dimples [44,45]. Thus, a near-spherical shaped mold would imprint deeper within the substrate and provide adequate dimple depth in spite of the spring-back effect. Moreover, the base of the near-spherical mold near the flat mold junction was kept wide enough to minimize stress concentration effects and potential mold failure. Cylindrical and rectangular molds are routinely used in nanoimprint lithography and the use of a near-spherical mold was chosen based on recent 3-dimensional molds for nanostructured free-form features in thermal nanoimprinting for practical applications [46,47]. The two layers of atoms (3000 atoms) of gold substrate at the bottom of the silicon substrate (along 8 nm height) had a fixed boundary condition. The temperature was held at 473 K and the mold was imparted a velocity of 50 m/s.

Molecular dynamic models (mold and substrate) employed periodic boundary conditions in the lateral plan (*x* and *y* axis) to replicate the experimental nanoimprint lithography (NIL) setup. The elemental interactions between the mold and the substrate were modeled using the Modified Embedded Atom Method (MEAM) potential [48]. The MEAM is a comprehensive potential to capture all the mold-substrate interactions within the NIL process. The MEAM potential values mentioned in Table 1 for gold (Au) and silicon (Si) were recalled from the library of MEAM potential parameters and these values were applied for Si-Au interaction as well as Au-Au interaction. In the MEAM formulation, the total energy E of a system of atoms is defined by Equation (1).

(1) $$E=\underset{i}{\sum }\left\{F_{i}\left(\bar{\rho_{i}}\right)+\frac{1}{2}\underset{i\neq j}{\sum }\varphi_{ij}\left(r_{ij}\right)\right\}$$

where, *F_i_* resembles the energy to embed an atom of type *i* into the background electron density at site *i*, *ρ_i_* represents the electron density, and *φ_ij_* represents the pair potential interaction between atoms *i* and *j* that is separated by *r_ij_*. The electron density is given by Equation (2).

(2) $$\rho_{i}=\frac{2{\rho_{i}}^{\left(0\right)}}{1+\exp\;[-\sum_{s=1}^{3}t_{i}^{\left(s\right)}\;{\left({\rho_{i}}^{\left(s\right)}/{\rho_{i}}^{\left(0\right)}\right)}^{2}}$$

where, the parameters *t_i_^(s)^* are weighting parameters for the atomic electron densities. *s* = 0,...,3, are partial electron densities defined by Equation (3).

(3) $${\left({\rho_{i}}^{\left(s\right)}\right)}^{2}=\sum_{j,k\neq i}\rho_{j}^{\alpha \left(s\right)}\;\left(r_{ij}\right)\;{\rho_{k}}^{\alpha \left(s\right)}\;\left(r_{ik}\right)L^{\left(s\right)}\;(\cos\theta_{jik})$$

where, the functions *L^(s)^(z)* are Legendre polynomials, *L^(0)^*(*z*) = 1, *L^(1)^*(*z*) = *z*, *L^(2)^*(*z*) = *z*^2^−1/3, *L^(3)^*(*z*) = *z*^3^−3/5*z*,α^(s*)*^ is the exponential decay factor. The electron density functions from atom *j* at a distance *r_ij_* from atom *i* are given by Equation (4).

(4) $$\rho_{j}^{\alpha \left(s\right)}\;\left(r_{ij}\right)=f_{c}\;\left(r_{ij}\right)f_{j}^{0}\;e^{-\beta_{j}^{\left(s\right)}}\;\left(r_{ij}/r_{j}^{0}-1\right)$$

here, $f_{j}^{0}$, $r_{j}^{0}$, and $\beta_{j}^{\left(s\right)}$ are parameters, *f_c_* is a cutoff function.

Table 1 shows, *α^0^* as the exponential decay factor for the universal equation of state; *β ^(^*^0−3)^ represent the exponential decay factors for the atomic electron densities; *t* ^(1−3*)*^ represent the weighting parameters for the atomic electron densities for both silicon mold and gold substrate, respectively [48].

A microcanonical (NVE where, N is the number of particles, V is the system volume, and E is the system energy) ensemble was used wherein a rescaling thermostat controlled the temperature of simulations by rescaling the velocities of the substrate atoms. The final temperature of 473 K was attained by ramping the temperature at equal intervals at every time step that was initialized from room temperature 293 K. The simulations were conducted at an integration time step of 1 femtosecond.

A five-step was applied to the nanoscale deformation process wherein the silicon mold was imprinted into the gold substrate. The first step included raising the temperature of the system for 50,000 time steps (to heat the system to 473 K); mold insertion for 78,000 time steps (to achieve a complete insertion of mold into the substrate); system cooling for 50,000 time steps (to lower the temperature to 293 K); relaxation of the system for 50,000 time steps (allowing rearranging of atoms at 293 K); and demolding for 160,000 time steps at velocity of 50 m/s (to completely disengage the mold from the substrate). The molding phase included both the mold insertion and lowering system temperature to room temperature. For the nanoimprint lithography process the mold insertion and removal phases impact the accuracy of the final geometry [29,49]. Thus, it is critical to understand the mechanics of these phases which can vary depending on the shape of the nanoimprint stamp. All six pressure tensors were recorded for the substrate atoms and thereby the von Mises stresses were calculated as shown in Equation (5).

(5) $$F_{vM}=\sqrt{\frac{{\left(p_{xx}-p_{yy}\right)}^{2\;}+{\left(p_{yy}-p_{zz}\right)}^{2\;}+{\left(p_{zz}-p_{xx}\right)}^{2\;}+6\times \left({p_{xy}}^{2}+{p_{xz}}^{2}+{p_{yz}}^{2}\right)}{2}}$$

The different components of the pressure tensor are given by p*_xx_*, p*_yy_*, p*_zz_*, p*_xy_*, p*_xz_*, and p*_yz_* whereas the von Mises stress is computed by F*_vM_*.

The effect of mold profile on the imprint quality was determined using a quantitative measure of spring back phenomenon. Percentage (%) spring back was calculated by Equation (6).

(6) $$\%\;Spring\;back=\frac{(L_{mold}-L_{demold})\times 100}{L_{mold}}$$

where, *% Spring back* is the spring back value in the horizontal or vertical direction, L*_mold_* is the initial dimension during molding, and L*_demold_* is the final dimension after demolding, respectively. A positive spring back indicates an expansion of the substrate shape (wider slot or deeper cavity), whereas a negative spring back indicates a contraction of the substrate shape (narrow slot or shorter cavity), in respective dimensions.

## 3. Results and Discussions

**Figure 2:**
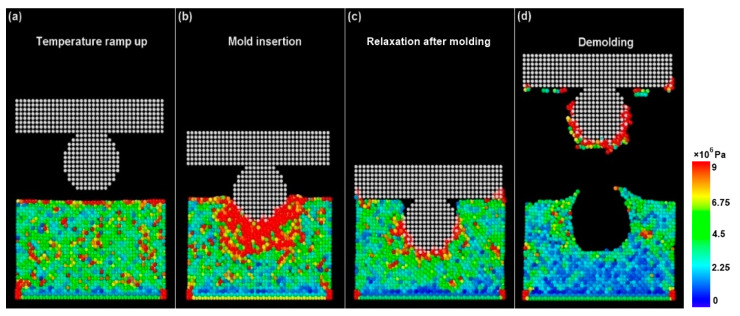
von Mises stresses during the NIL process using a spherical mold profile. Time steps for each phase of NIL: (**a**) 50,000, (**b**) 38,000, (**c**) 50,000, and (**d**) 160,000.

The effect of varying mold pattern designs on the deformation behavior and the von Mises stresses was investigated through molecular modeling. Figure 2 represents the von Mises stresses during the NIL process using a spherical mold profile with an aspect ratio of 1. The other mold types including rectangular and cylindrical also had an aspect ratio of 1 to aid consistency while evaluating the results. Per atom von Mises stress was calculated at each stage of the NIL process. Figure 2a shows that the temperature is ramped up to 473 K in uniform increments. Residual von Mises stresses are exhibited due to an increase in the temperature causing motion of the gold atoms. As the spherical mold is inserted in the gold substrate there is a rapid increase in the von Mises stresses. These stresses reach their peak value during the initial penetration stage of the mold. Figure 2b clearly shows substantially higher stress levels (1.08 × 10^7^ Pa) experienced by atoms in the close vicinity of the mold. Figure 2c shows the relaxation of gold atoms after complete penetration of the mold within the substrate. Herein, the temperature of the system is slowly ramped down to 293 K allowing dislocation of the gold atoms around the mold geometry. In the final phase of demolding shown in Figure 2d, the mold is retrieved from the gold substrate leaving a spherical imprint with the lowest von Mises stresses (1.2 × 10^6^ Pa).

**Figure 3:**
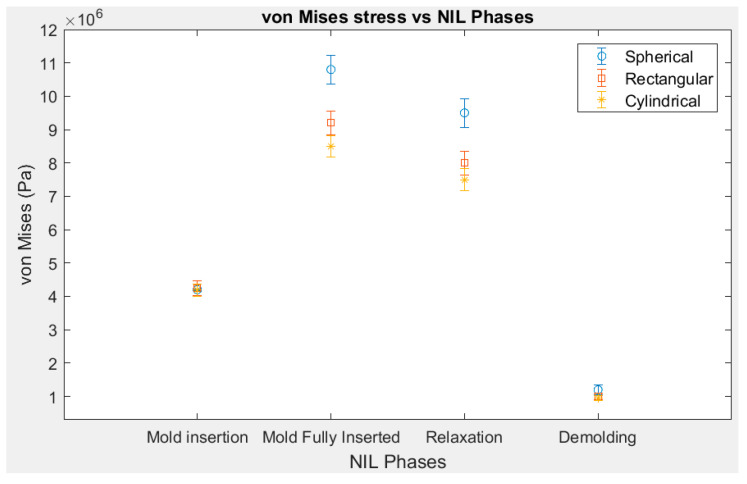
von Mises stresses at various NIL phases for different mold profiles. The time steps are initial mold insertion—20,000; mold fully inserted—58,000; relaxation—50,000; demolding—160,000.

Figure 3 show the average von Mises stress values of the substrate at a particular frame/time step. The error bar represents variation of the von Mises stresses within the substrate. Figure 3 depicts the analogous behavior for rectangular and cylindrical mold profiles. For the rectangular and cylindrical mold profiles, the maximum stress values recorded were 9.20 × 10^6^ Pa and 8.50 × 10^6^ Pa, respectively. Thus, among three mold profiles the spherical mold profile recorded the highest von Mises stress. This is due to the highest contact surface area of the hemispherical region of the spherical mold that was pressed against the gold substrate during the mold insertion phase. The von Mises stresses for the rectangular and cylindrical molds were much lower as their contact surface areas during imprinting were 36% and 50% smaller in size respectively, in comparison to the spherical stamp.

**Figure 4:**
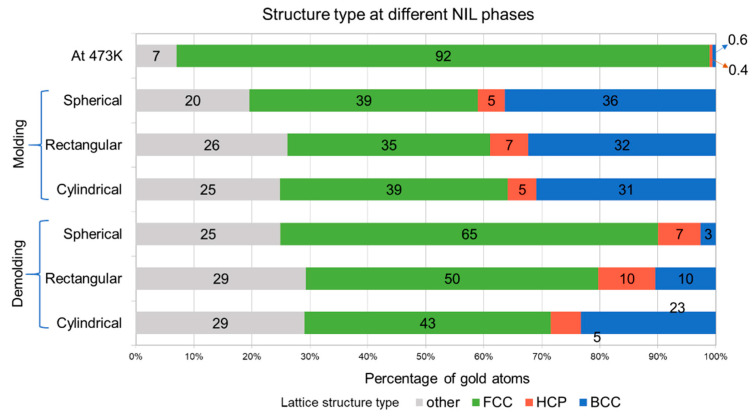
Lattice deformation of gold substrate with different mold profiles. Time steps for each phase are: Raise temp to 473 K—50,000; Molding—78,000; Demolding—160,000.

**Figure 5:**
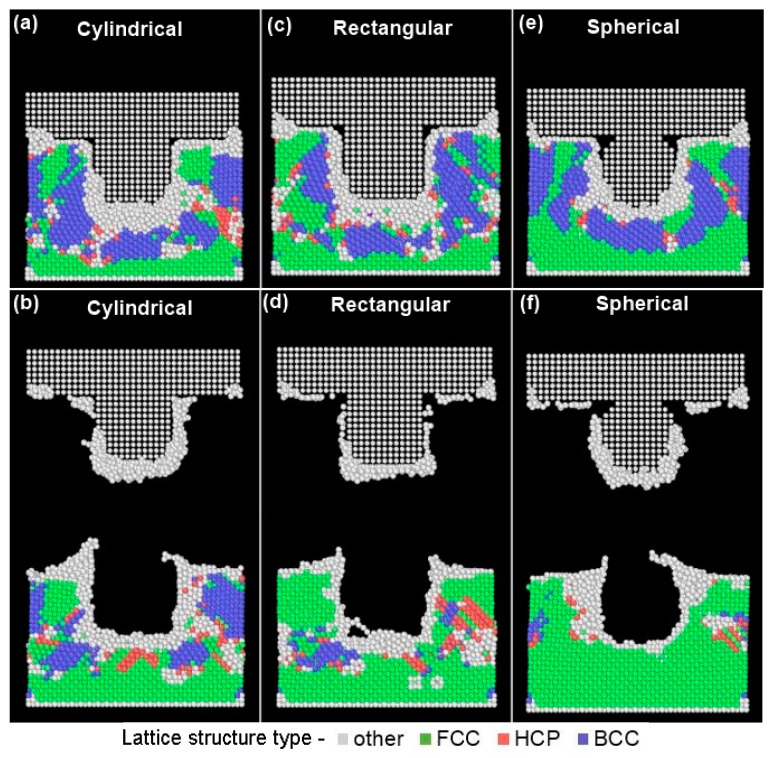
Lattice deformation behavior after molding (top row) and mold removal (bottom row) for different types of mold patterns. Time steps for (**a**,**c**,**e**)—78,000; and (**b**,**d**,**f**)—160,000.

The lattice deformation of the gold substrate was evaluated at different stages of the imprinting process. A Polyhedral Template Matching (PTM) method [50] was used to study the crystal structure of gold across all NIL phases. The key local crystalline structures investigated included Face-Centered Cubic (FCC), Hexagonal Closed Packed (HCP), and Body-Centered Cubic formed which occurred as the gold substrate underwent deformation. The PTM method evaluates the neighborhood atoms and is not affected by thermal variations. A root mean square deviation (RMSD) cutoff value of 0.15 was used for the PTM analysis. This RMSD value performs better to study the crystalline nature of a solid and can assist in investigating the deformation behavior. Different crystalline structures including FCC, BCC, and HCP were identified for the gold atoms based on variation in their topographies using this cutoff. Lattice structure labelled as “other” included deformed or spurious atomic structures. Defects were also captured using Isohedral Coordination (ICO) and Simple Cubic (SC) structures. Figure 4 and Figure 5 depict the variation in lattice behavior using the initial penetration and mold removal phases for the three different mold profiles. All the structure types defined herein were computed using LAMMPS and matched with the PTM analysis to identify distinct lattice placements. Figure 4 shows that after ramping the temperature to the recrystallization temperature (473 K) there is a lattice rearrangement reducing the FCC structure to 92% with spurious structures occupying 7% of the total lattice structure. After the molding phase, the original FCC structure of gold atoms underwent deformation into hexagonal closed pack, body centered cubic, and other structures. BCC atoms composed of 31%, 32%, and 36% for cylindrical, rectangular, and spherical molds, respectively. This transformation can be attributed to the Bain path consisting of the dilation of FCC gold substrate atoms in the x and y-direction due to the movement of gold atoms away from the mold insertion area [51]. This phenomenon is accompanied by a contraction in the z-direction during the molding phase which occurs due to the downward movement of the mold into the gold substrate [50,52]. While deforming along the Bain path, the system becomes tetragonally distorted wherein, the system changes its volume in at least two directions. The periodic nature of the x and y lattices permits distortion towards phase transition.

Figure 5 shows mold penetration (top row) and mold retrieval (bottom row) for all the mold geometries. All the mold types show the presence of “other” structure types around the periphery of the mold profile (Figure 5-top row). This is because, as the molds penetrate within the gold substrates the atoms in the vicinity of the mold undergo high-strain plastic deformation by slip which increases the misorientation with more dislocations trapped within cell interiors thereby losing their FCC structure [53]. Similar phenomena are observed for gold atoms directly below the mold profile. Our work is unique to metallic nanoimprint lithography and analogous findings are supported in the literature for adhesion of polymeric systems to mold surfaces [54]. Figure 5b clearly shows that the substrate with cylindrical mold had a cleaner imprint as compared to the rectangular mold due to smoother transition of the mold profile. The rectangular mold has orthogonal sides which lead to abrupt detachment and tearing of the gold substrate atoms as shown in Figure 5d. The spherical mold (Figure 5f) also shows a relatively smoother substrate profile.

In the mold retrieval phase, the gold atoms regained their lattice structure of FCC atoms to 43%, 50%, and 65% for cylindrical, rectangular, and spherical molds, respectively. This was complemented by a reduction in the BCC atoms to 23%, 10%, and 3% from 31%, 32%, and 36% for cylindrical, rectangular, and spherical molds, respectively. Figure 5 (bottom row) show that gold atoms adhere to the silicon mold as it is completely retrieved from the substrate after the demolding phase of NIL. This can be attributed to the higher surface energy [55] between the gold substrate atoms and the silicon mold tooth forming weldments during the cooling and relaxation phases of the NIL process.

During the mold retrieval phase the gold atoms had expansion along the z-dimension and this behavior was also confirmed by studies in the literature [35,56,57]. During the demolding process, the spherical mold has the highest portion of the FCC structure due to the tensile stresses applied by the retrieving mold in the vertical direction. This transformation can be attributed to the Bain path consisting of the dilation of FCC gold substrate atoms to the vertical (*z-*direction) from the *x* and *y*-direction due to the movement of gold atoms away from the mold insertion area [48].

**Figure 6:**
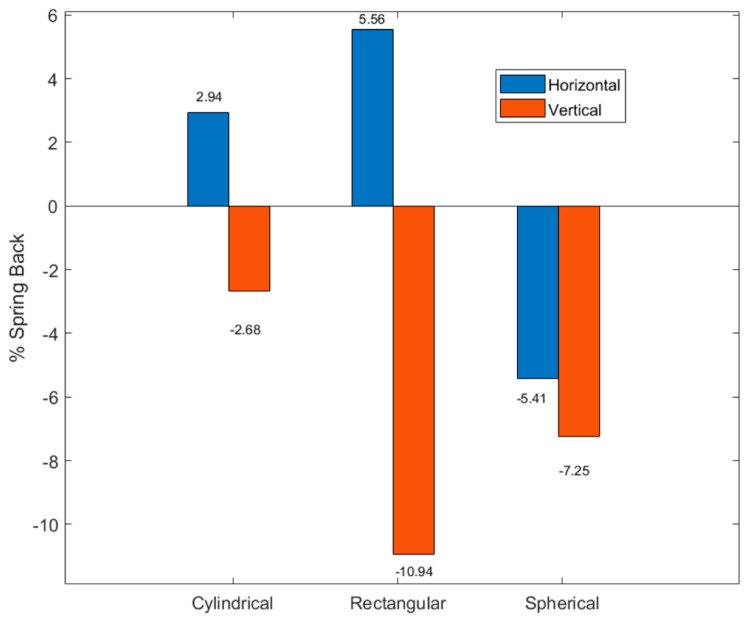
% Spring back for the gold substrate imprinted with different mold profiles at respective directions.

Figure 6 shows the percentage spring back for the gold substrate imprinted with different mold profiles at respective directions. This measure was implemented to quantify the quality of the imprint. A positive spring back indicated an expansion of the substrate dimensions “cavity” in respective directions, whereas a negative spring back indicated contraction of the cavity dimension. A high-quality imprint occurs when the variation of spring back is minimal in either or both directions. The substrate imprinted with the cylindrical mold displayed the lowest spring back effect in both the horizontal and vertical directions. Thus, the cylindrical mold had the highest quality imprint as compared to the rectangular and spherical molds, respectively. The rectangular mold had the largest vertical spring back (−10.94%) followed by the spherical mold (−7.25%). This can be confirmed by Figure 5d wherein substrate atoms are sheared and dislodged from the bottom of the cavity. Moreover, the substrate with the rectangular mold has a wider cavity in the horizontal direction (% spring back = 5.56%) as compared to the mold profile. Figure 5f clearly shows that the spherical cavity in the substrate has smaller dimensions as compared to the mold dimensions in both the horizontal (−5.41%) and vertical (−7.25%) directions.

An important finding of this research is that the gold substrate eventually retains its FCC structure at the end of the nanoimprint lithography process. Thus, the ductility of the material is maintained based on the heating and cooling cycles which results in a high-quality imprint for the cylindrical mold as compared to the other mold types. Future research can explore the use of anti-stick components on the mold profiles to evaluate their impact on the nanoimprinting process.

## 4. Conclusions

Different silicon mold profiles were implemented to study their effect on the deformation behavior of gold using molecular dynamics simulations. Von Mises stress, lattice dislocations, and material deformation were investigated for cylindrical, rectangular, and spherical mold profiles. Relatively higher von Mises stress was observed for the spherical mold profile compared to the rectangular and cylindrical profiles due to the larger surface area of contact in the initial mold penetration stage. However, significant reductions in von Mises stresses were observed during the cooling and mold retrieval phases. The high strain deformation during the mold penetration resulted in conversion of FCC structures to BCC. Finally, the slow cooling and retrieval of mold from the gold substrate had an impact on retaining the FCC structure as well as a high-quality imprint profile. The gold substrate with the cylindrical mold imprint had the lowest percentage spring back in both the horizontal and vertical directions thereby, replicating the mold profile as compared to other mold types. This research forms the basis for the development of design guidelines of nanoimprint molds with cylindrical, rectangular, and spherical mold profiles.

## Figures and Tables

**Table 1:** Physical properties and MEAM potentials for gold and silicon [48].

Element	Lattice Constant (Å)	Melting Point (K)	Density (kg/m^3^)	* t^(1)^*	* t^(2)^*	* t^(3)^*	* α^0^*	* β^0^*	* β^1^*	* β^2^*	* β^3^*
Silicon	5.431	1687	19300	3.30	5.10	−0.80	4.87	4.8	4.8	4.8	4.8
Gold	4.065	1337	2328	1.58	1.50	2.60	6.34	5.45	2.2	6	2.2

## Data Availability

The data presented in this study are available on request from the corresponding author.

## References

[1] Chou S.Y., Krauss P.R., Renstrom P.J. (1996). Nanoimprint lithography. J. Vac. Sci. Technol. B Microelectron. Nanometer Struct..

[2] Gaikwad A. (2020). Understanding Material Deformation Mechanism in Nanoimprint Lithography for Engineering and Medical Applications. Ph.D. Thesis.

[3] Chou S.Y. (1997). Sub-10 nm imprint lithography and applications. J. Vac. Sci. Technol. B Microelectron. Nanometer Struct..

[4] Pease R.F., Chou S.Y. (2008). Lithography and Other Patterning Techniques for Future Electronics. Proc. IEEE.

[5] Chou S.Y., Krauss P.R. (1997). Imprint lithography with sub-10 nm feature size and high throughput. Microelectron. Eng..

[6] Ko S.H., Park I., Pan H., Grigoropoulos C.P., Pisano A.P., Luscombe C.K., Frechet J.M.J. (2007). Direct Nanoim printing of Metal Nanoparticles for Nanoscale Electronics Fabrication. Nano Lett..

[7] Buzzi S., Robin F., Callegari V., Löffler J.F. (2008). Metal direct nanoimprinting for photonics. Microelectron. Eng..

[8] Sealy C. (2005). Road map to nanomanufacturing: Nanofabrication. Mater. Today.

[9] Sun H., Yin M., Wang H. (2017). High Aspect Ratio Nanoimprint Mold-Cavity Filling and Stress Simulation Based on Finite-Element Analysis. Micromachines.

[10] Tada K., Hirai Y. (2010). Molecular Dynamics Study on Mold and Pattern Breakages in Nanoimprint Lithography. Lithography.

[11] Jiang W., Ding Y., Liu H., Lu B., Shi Y., Shao J., Yin L. (2008). Two-Step curing method for demoulding in UV nanoimprint lithography. Microelectron. Eng..

[12] Quang-Cherng H., Chen-Da W., Te-Hua F. (2004). Deformation Mechanism and Punch Taper Effects on Nanoimprint Process by Molecular Dynamics. Jpn. J. Appl. Phys..

[13] Pei Q.X., Lu C., Liu Z.S., Lam K.Y. (2007). Molecular dynamics study on the nanoimprint of copper. J. Phys. D Appl. Phys..

[14] Yuan Y., Sun T., Zhang J., Yan Y. (2011). Molecular dynamics study of void effect on nanoimprint of single crystal aluminum. Appl. Surf. Sci..

[15] MacIntyre D., Thoms S. (2005). A study of resist flow during nanoimprint lithography. Microelectron. Eng..

[16] Hsiung Y.H., Chen H.Y., Sung C.K. (2009). Temperature effects on formation of metallic patterns in direct nanoimprint technique—Molecular dynamics simulation and experiment. J. Mater. Process. Technol..

[17] Odujole I.J., Desai S. (2020). Molecular dynamics investigation of material deformation behavior of PMMA in nanoimprint lithography. AIP Adv..

[18] Odujole J., Desai S. (2020). Atomistic Investigation of Material Deformation Behavior of Polystyrene in Nanoimprint Lithography. Surfaces.

[19] Odujole J., Desai S. Molecular Dynamics Simulation of Poly Acrylic Acid as a Resist Material for Thermal Nanoimprint Lithography Processes. Proceedings of the Industrial Engineers Research Conference.

[20] Akter T., Desai S. (2018). Developing a predictive model for nanoimprint lithography using artificial neural networks. Mater. Des..

[21] Almakaeel H., Albalawi A., Desai S. (2018). Artificial neural network based framework for cyber nano manufacturing. Manuf. Lett..

[22] Cordeiro J., Desai S. (2016). The Leidenfrost Effect at the Nanoscale. J. Micro Nano Manuf..

[23] Hollingsworth S.A., Dror R.O. (2018). Molecular Dynamics Simulation for All. Neuron.

[24] Rodrigues J., Desai S. (2019). The nanoscale Leidenfrost effect. Nanoscale.

[25] Marquetti I., Desai S. (2019). Orientation effects on the nanoscale adsorption behavior of bone morphogenetic protein-2 on hy-drophilic silicon dioxide. RSC Adv..

[26] Marquetti I., Desai S. (2018). Molecular modeling the adsorption behavior of bone morphogenetic protein-2 on hydrophobic and hydrophilic substrates. Chem. Phys. Lett..

[27] Marquetti I., Rodrigues J., Desai S.S. (2018). Ecological Impact of Green Computing Using Graphical Processing Units in Molecular Dynamics Simulations. IJGC.

[28] Cordeiro J., Desai S. (2017). The Effect of Water Droplet Size, Temperature, and Impingement Velocity on Gold Wettability at the Nanoscale. J. Micro Nano Manuf..

[29] Gaikwad A., Clarke J. Molecular Dynamics Study of the Quenching Effect on Direct Nanoimprint of Gold. Proceedings of the 2019 IISE Annual Conference.

[30] Zhang J., Zhang Y., Mara N., Lou J., Nicola L. (2014). Direct nanoimprinting of single crystalline gold: Experiments and dislocation dynamics simulations. Appl. Surf. Sci..

[31] Lebreton C., Wang Z. (1996). Nanofabrication on gold surface with scanning tunneling microscopy. Microelectron. Eng..

[32] Gaikwad A., Desai S. (2018). Understanding Material Deformation in Nanoimprint of Gold using Molecular Dynamics Simulations. Am. J. Eng. Appl. Sci..

[33] Gaikwad A., Odujole J., Desai S. (2020). Atomistic investigation of process parameter variations on material deformation behavior in nanoimprint lithography of gold. Precis. Eng..

[34] Kontio J.M., Husu H., Simonen J., Huttunen M.J., Tommila J., Pessa M., Kauranen M. (2009). Nanoimprint fabrication of gold nanocones with 10 nm tips for enhanced optical interactions. Opt. Lett..

[35] Manson S.S., Halford G.R. (2009). Fatigue and Durability of Metals at High Temperatures.

[36] Plimpton S. (1995). Fast Parallel Algorithms for Short-Range Molecular Dynamics. J. Comput. Phys..

[37] Alexander S. (2010). Visualization and analysis of atomistic simulation data with OVITO—The Open Visualization Tool. Model. Simul. Mater. Sci. Eng..

[38] Towns J., Cockerill T., Dahan M., Foster I., Gaither K., Grimshaw A., Hazlewood V., Lathrop S., Lifka D., Peterson G.D. (2014). XSEDE: Accelerating Scientific Discovery. Comput. Sci. Eng..

[39] Lee S., Park S., Cho D.-I. (1999). The surface/bulk micromachining (SBM) process: A new method for fabricating released MEMS in single crystal silicon. J. Microelectromech. Syst..

[40] Rao A.V.N., Pal P., Pandey A.K., Menon P.K., Tanaka H., Sato K. High Speed Silicon Wet Bulk Micromachining of Si {111} in KOH Based Solution. Proceedings of the 2020 Symposium on Design, Test, Integration & Packaging of MEMS and MOEMS (DTIP).

[41] Rao A.V.N., Swarnalatha V., Pandey A.K., Pal P. (2018). Determination of precise crystallographic directions on Si {111} wafers using self-aligning pre-etched pattern. Micro Nano Syst. Lett..

[42] Feng Y., Yang X., Zhang Z., Kang D., Zhang J., Liu K., Li X., Shen J., Wang T. (2019). Epitaxy of single-crystalline GaN film on CMOS-compatible Si (100) Substrate buffered by graphene. Adv. Funct. Mater..

[43] Ryou J.-H., Yoder P.D., Liu J., Lochner Z., Kim H., Choi S., Kim H.J., Dupuis R.D. (2009). Control of quantum-confined stark effect in InGaN-based quantum wells. IEEE J. Sel. Top. Quantum Electron..

[44] Wagoner R.H., Lim H., Lee M.-G. (2013). Advanced Issues in springback. Int. J. Plast..

[45] Wu C.-D., Li R.-E. (2020). Effects of alloy composition, cavity aspect ratio, and temperature of imprinted ZrCu metallic glass films: A molecular dynamics study. Appl. Phys. A.

[46] Kang M.G., Kim M.-S., Kim J., Guo L.J. (2008). Organic solar cells using nanoimprinted transparent metal electrodes. Adv. Funct..

[47] Wu J., Lee W.L., Low H.Y. (2019). Nanostructured Free-Form Objects via a Synergy of 3D Printing and Thermal Nanoim-printing. Glob. Chall..

[48] Baskes M.I. (1992). Modified embedded-atom potentials for cubic materials and impurities. Phys. Rev. B.

[49] Xia W., Su Y., Li T., Liu C., Li D., Duan Z. (2013). Study of demolding in nanoimprint lithography with pseudoplastic metal nanoparticle fluids. RSC Adv..

[50] Larsen P.M., Schmidt S., Schiøtz J. (2016). Robust structural identification via polyhedral template matching. Model. Simul. Mater. Sci. Eng..

[51] Goubet N., Pileni M.-P. (2013). Negative supracrystals inducing a FCC-BCC transition in gold nanocrystal superlattices. Nano Res..

[52] Zhang X., Sun C., Fang N. (2004). Manufacturing at Nanoscale: Top-Down, Bottom-up and System Engineering. J. Nanopart. Res..

[53] Gray G.T. (2012). High-Strain-Rate Deformation: Mechanical Behavior and Deformation Substructures Induced. Annu. Rev. Mater. Res..

[54] Perumal J., Yoon T.H., Jang H.S., Lee J.J., Kim D.P. (2009). Adhesion force measurement between the stamp and the resin in ultraviolet nanoimprint lithography—An investigative approach. Nanotechnology.

[55] Wang Y.M., Cai W. (2015). Evaluation of the Surface Tension of Silicon-Gold Binary Liquid Alloy. Mater. Sci. Forum.

[56] Engin C., Urbassek H.M. (2008). Molecular-dynamics investigation of the fcc → bcc phase transformation in Fe. Comput. Mater. Sci..

[57] Marder M.P. (2010). Condensed Matter Physics.

